# A scoping review of health systems resilience assessment frameworks

**DOI:** 10.1371/journal.pgph.0003658

**Published:** 2024-09-23

**Authors:** Sushma Marita Dsouza, Anuradha Katyal, Shrikant Kalaskar, Mehnaz Kabeer, Latika Rewaria, Sreemeena Satyanarayana, Krishna Reddy Nallamalla, Maulik Chokshi

**Affiliations:** 1 ACCESS Health International, India; 2 Sambodhi Research & Communications Pvt. Ltd., Noida, Uttar Pradesh, India; Dalhousie University, CANADA

## Abstract

Health system resilience is a prerequisite for effectively managing cataclysmic events adversely affecting health outcomes. The COVID-19 pandemic reasserted the importance of having resilient health systems and called for a relook at the existing framework that measures health system resilience. Mixed methods were used in this study. The review started with the measurement of health systems resilience and its context. Ebola epidemic triggered the importance, hence our search focused on published literature from 2014 to 2021. Based on the review, a semi-structured tool was developed for key in-depth interviews of seven experts from different countries. The frameworks focused on climate change, disaster management, health systems, city-specific resilience, and e-resilience were reviewed. In-depth interviews highlighted that resilient health systems need to engage the private sector, priority areas like leadership and governance, health resources, and a unified agenda for global collaboration. From experts’ point of view, the inherent nature of health systems to respond to shock was clearly defined as the resilient health system. Health systems resilience definition needs to be defined, based on which assessment indicators will be identified. Indicators need to evolve continuously and be able to measure resilience at sub-national, national, regional, and global levels.

## Introduction

The concept of resilience emerged in ecology in the 1970s following the work of ecologist C.S. (Buzz) Holling. According to Holling, resilience is “*a measure of the persistence of systems and their ability to absorb change and disturbance while maintaining the same relationships between populations or state variables*.” [1] This concept was quickly applied in psychology, where early research primarily focused on trauma-affected individuals. Discussions on livelihood approaches have emphasized the role of social determinants of health in building resilience and this marked the inception of resilience-related research within the field of health systems [2]. The concept of resilience in Health Systems gained traction following the emergence of natural and manmade disasters. These include disease outbreaks like coronaviruses (SARS CoV and SARS Cov-2), Human Immunodeficiency Virus Infection/Acquired Immune Deficiency Syndrome (HIV/AIDS), Ebola virus disease, Zika virus, and the Middle East Respiratory Syndrome (MERS) and disasters like the Tsunami and Haiti earthquake [3]. It was then purported that all institutions and resources aiming to improve health are integral components of health systems and play a role in ensuring its resilience. The health system resilience concept paved a way for cross-sector collaboration and enhancing the non-medical determinants to making health systems resilient [4].

Following the West African Ebola epidemic in 2014–15, resilience was further recognized as an essential attribute for strong health systems[5]. The countries of Western Africa had limited capacity during the Ebola outbreak [6]. Several challenges were observed in these countries like the contacts of confirmed cases were not systematically identified, monitored, and diagnosed early. Additionally, new cases emerging in previously unaffected communities were not quickly diagnosed, and isolated [7]. Western African countries did not engage in global cooperation initially. However, the importance of global collaboration and the reinforcement of surveillance measures were subsequently emphasized.

The recent COVID-19 pandemic revealed similar alarming gaps in disease surveillance in several parts of the world [8]. Even the health systems that had attained Universal Health Coverage were unable to provide the required response during the pandemic due to unexpected surge and scale [9].

Given the growing importance of Health system resilience, delineating measures to assess it, could potentially enhance the preparedness, resource optimization, and learning and adoption of change. In this paper we intend to estimate and understand how resilience is being measured world over and what are the different frameworks which exist.

## Methods

We used an exploratory research design for our scoping review. The aim was to review and understand the evolution of various frameworks for health system resilience. This also included identifying various frameworks used for assessing health system resilience and understanding their elements. The review was further strengthened with peer-to-peer discussions and in-depth interviews with experts.

### Data collection process

#### Secondary data collection

The review was initiated by searching articles from PubMed and Google scholar. The review included articles published in the English language only. During the literature search, the following search terms were used: (health system OR healthcare OR health care system) AND (resilience OR resilient) AND (framework). The first stage of selection and screening was done after reviewing the title and abstract of the papers for the inclusion of the term “resilience”. Secondly, the full-text review of selected papers was done, if they met the inclusion criteria. The articles were included based on these criteria: (a) Articles published from 2014 to 2021; (b) the frameworks should be applicable globally; (c) they should have measurable indicators; (d) they should have a discernible relationship with health care; (e) Frameworks published by reputed institutions e.g.: WHO, USAID, Johns Hopkins, etc., or in a peer reviewed journal. Additionally, we performed the manual search of citations from the relevant articles.

**Fig 1:**
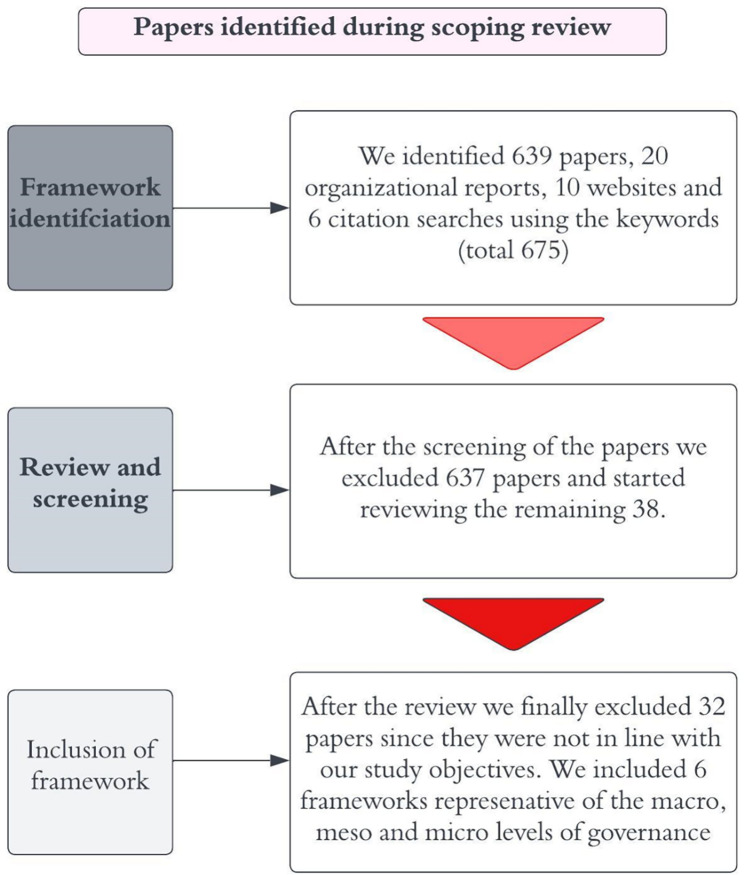
Scoping review approach.

A total of 639 studies were selected through the database search, 30 articles were selected from websites and 10 articles were selected from organizations and 6 articles were selected from citation search. The 639 articles were screened for title and abstract. Screening of title and abstract resulted in exclusion of 604 irrelevant articles. A total of 30 articles were assessed for full text screening, out of which 24 articles were excluded with reasons such as not relevant to the study (6), not relevant to the collaborative (10) and no tangible framework suggested (11). We finalized a total 6 studies that were eligible for final review (Fig 1).

#### Primary data collection

In-depth interviews with experts and peer to peer learning workshops within the field of health systems resilience from across seven countries. The interviews were conducted using a semi-structured interview guide, and interviews were audio recorded after obtaining informed verbal consent from participants. Insights from these interviews helped us gain diverse perspectives related to health systems resilience in their respective countries.

## Results and findings

### Findings from the secondary research

The secondary research helped us identify six distinct frameworks (Table 1) from within the literature pertaining to health systems resilience. These frameworks discuss the resilience of health systems at various levels, including the micro level (health facilities, cities, and sub-provincial regions), the meso level (provincial) and the macro level (national and international contexts).

**Table 1:** Frameworks reviewed in the study.

Framework Name	Year	Description	Scale of measurement	Measures
Disaster Mitigation Framework	2016	This is a macro, meso and micro level framework used to increase risk awareness and promote resilience by promoting the usage of mitigation enhancing products, services and assets.	Central, provincial and local governments Private and public sector entities Communities, families and individuals	The framework explores seven core capabilities required for entities involved in mitigation: threats and hazards identification, risk and disaster resilience assessment, planning, community resilience, public information and warning, long-term vulnerability reduction, and operational coordination.
UN’s Sendai Framework for Disaster Risk Reduction (SFDRR) and sustainable development goals (SDGs)	2017	As a successor to UN’s earlier frameworks on disaster risk reduction the SFDRR focused on health. It was based the proposition that resilient health systems as an opportunity for ensuring effective DRR in the health sector. It was in response to several disasters pre-2015, both man-made and natural.	Health System	Disaster risk reduction (DRR), Disaster preparedness, Disaster response and Post-disaster recovery are mapped against Health leadership and governance, Health financing, Medical products, vaccines, and technologies, Health information management, Human resources for health (HRH) and Service Delivery
HS - WHO	2020	A macro level review of the various phases of a pandemic and how countries (mostly European) have responded across various domains and sub domains of a health system. It encompasses a wide range of assessment areas that can be applied to various aspects of health system resilience	Health System/Country	This framework delineates various indicators against—Governance, Financing, Resources and Service Delivery; and stratifies them between the phases of preparedness, shock and recovery
Checklist to improve health system resilience to infectious disease outbreaks and natural hazards	2020	Identifies ten thematic categories identified in our research as important components of resilient health systems, including core health system capabilities/capacities, infrastructure/transportation, financing, barriers to care and communication, surge capacity, risk communication, workforce and infection control.	Health facility and health officials	A health facility is assessed for existence of certain processes like a system for reporting, Sufficient financing, protocols for hazardous waste, plans for distributing emergency, established relationships with community, plans for coordinating with other facilities, established leadership hierarchy, represented at Emergency Operations Center, and support workforce training.
City Resilience by Rockefeller Foundation	2015	A city-based framework which looks at disaster and climate change with health being one of the social causes which would need attention, City resilience describes the capacity of cities to function, so that the people living and working in cities–particularly the poor and vulnerable–survive and thrive no matter what stresses or shocks they encounter.	Sub national—urban	Framework discusses Leadership & Strategy, Health & Wellbeing, Economy & Society and Infrastructure & Environment as the key categories to distinguish a resilient city from one that is simply liveable, sustainable or prosperous
E-resilience	2020	The United Nations Economic and Social Commission for Asia and the Pacific (ESCAP) has been promoting e-resilience and inclusive broadband as part of the regional broadband connectivity initiative.	Various sectors of a country	Discusses (Information Communication Technology) ICT for health and social resilience, it’s role in setting up new systems and applications, ICTs’ role in data management and ICT infrastructure as a physical foundation for the Risk Prevention, Risk Reduction, Preparedness, adaptation and Response and the Recovery Phase

Source: [4,10–14].

We began by reviewing the Disaster Mitigation Framework by the US government which clearly defines mitigation roles across the entire community [10] The 2017 UN Sendai Framework for Disaster Risk Reduction (SFDRR) and Sustainable Development Goals (SDGs) strongly recommend implementing disaster risk reduction (DRR) strategies to improve global resilience against disasters [4]. Unlike its predecessors the SFDRR places a stronger emphasis on resilient health systems for disaster risk reduction.

Thirdly, the 2020 Health Systems—World Health Organization maps the essential health system building blocks and stages in the shock cycle. It not only provides distinctions among the building blocks but also reiterates the overlaps across stages and strategies [11].

At the facility level, developed a Health Systems Resilience Checklist, designed to identify the necessary capacities and capabilities crucial for health systems resilience during disaster [12].

The 2015 City Resilience Framework, developed by The Rockefeller Foundation consists of 12 key goals (one of which pertains to health) that define the essential outcomes of a resilient city,including public health systems, quality healthcare, medical care, and emergency response [13].

The role of e-resilience has been recognized as prerequisite to a resilient health system An analytical paper from the UN Economic and Social Commission for Asia and the Pacific (ESCAP) in 2020 discusses a framework for e-resilience, discusses the role of ICT in all the four phases of a pandemic: prevention, risk reduction, preparedness, adaptation and response, and the recovery [14].

### Findings from expert interviews

The thematic areas identified through expert interviews and peer-to-peer learning workshops are outlined below.

### Definition of resilience and scope of resilience

The definition of health systems resilience delineated by the participants included some key elements such as the ability of the system to identify, respond to, control, and manage the impact of the stress; the ability to immediately reconfigure when the next external shock is applied; systems’ capacity for absorption, adaptation, and transformation; ability to anticipate shocks and prepare for them (Table 2).

**Table 2:** Definitions of health systems resilience.

Respondent identity	Definitions
Academic Expert with Disaster response and Health system resilience experience	The ability to be resilient can primarily be seen in one’s capacity for absorption, adaptation, and transformation, but these abilities can also be attributed to a wide range of other, unrelated abilities.
Public Health Expert and Academician	Whenever there are stressors, forces, disasters and epidemics, the system will remain strong and be able to accomplish its goals as a system.
Physician and Health Economic Expert	The resilience of the health system is a reflection of the ability of the health system to respond to new threats or traditional stress, but in different ways. It is the ability of the system to identify, respond, control and manage the impact of the stress
Development partner and multi-country health financing expert	Resilience very much embodies a dynamic system, one that is able to maintain a steady-state when necessary, but is able to immediately reconfigure, when the next external shock is applied to each; to reconfigure in a way that whether the shock is to the system itself, or the system is being used to respond to that shock
Expert in Health system surveillance	A resilient health system refers to its capacity to withstand shocks. It is the ability to anticipate shocks and prepare for them, even if the plan is not implemented.
Academician and health systems expert	Health system resilience is all about how a system that is already existing can adapt and adjust to the evolving situation.
Academician with multi-country experience	Health system resilience is the ability of the health system to absorb surges in healthcare needs, that could be sudden or gradual, depending on how we define sudden and gradual.

### Resources

Many nations are confronted not only with shortages of human resources, essential vaccines and pharmaceutical interventions but also with challenges in rapidly expanding their infrastructure and training human resources for vaccine delivery. The systems need to harness existing local resources, complemented by external support, to develop solutions. In addition, many resource based indicators need to be repurposed for a disaster to ensure that the surge is met.

“*The conventional indicators of health systems and benchmarks such as human resources and supply chain are laid down during normal times*. *However*, *there is a need to redefine the indicators and benchmarks in terms of their applicability at the time of a surge in demand*.*”-* Health system resilience expert with focus on low resource settings.

### Priorities for ensuring health systems resilience

Participants mentioned the significance of adopting a “One Health” approach to address the potential risks originating from the interface of animal-human-ecosystems. This approach refers to the implementation of a collaborative and multidisciplinary strategy that promotes synergistic partnerships aimed at achieving shared public health goals. The discussion also delved into the crucial role of the community and emphasized the need to institutionalize real-time coordination and information generation mechanisms. A profound understanding of the social and cultural intricacies within the community is of important when undertaking initiatives related to health systems resilience.

“*We need to strengthen the global community to better coordinate different countries*, *sectors*, *and public-private sector collaborations in terms of financing and manufacturing medical equipment”-* Physician and Health Economic Expert.

### Engaging the private sector in mixed health systems

Service delivery must remain uninterrupted during times of crisis, in health systems that are resilient. This calls for engaging all the actors involved in health provisioning. Participants in the current study asserted that the private sector has been a key stakeholder in combating the any disaster and ensuring uninterrupted health services. However, there is a need to bolster the regulatory systems to ensure financial accountability and care quality.

### Cross- learning between countries and unified agenda

Participants stated that instead of focusing on one single country, we must explore what each of us can learn from each other. Different lessons come out of different countries that manage to deal with shocks and exhibit resilience in certain areas. Moreover, the learning can extend to what these countries could do in order to be more resilient.

Each country should consider applying lessons from other countries that displayed better pandemic control.

Collaboration between all nations is necessary for readiness, response, recovery, and mitigation of global health emergencies. These collaborations should go beyond employing the same methods and strategies to address global health emergencies. The participants mentioned the need to strengthen the data sharing aspect across the nations to promote global collaboration.

“*Sharing knowledge and providing technical assistance from one country to other countries will be beneficial for a coordinated pandemic response.”-* Health system resilience expert with focus on low resource settings

### Social determinants of health

Participants stressed the importance of considering elements outside the health systems to ensure the resilience of health systems. To interact with other systems that contribute to the resilience of the health system, resilience measures must extend beyond the healthcare industry. It is crucial to understand how health systems are influenced by other sectors, including education, food, travel, trade, and social protection. Defining resilience measures that address this dynamism can make it easier to discern the capacity for resilience across various systems.

“*A crucial aspect of resilience is the establishment of social protection mechanisms, particularly during pandemic spikes, when numerous essential priorities of the population remain unmet, leading to heightened poverty levels. It is imperative to identify the marginalized segments of the population and provide them with adequate support to prevent an increase in mortality rates resulting from the upsurge in poverty”-* Public Health Expert and Academician

### Frameworks for health systems resilience

Thet participants stated that to better conceptualize health systems resilience a framework can be instrumental in determining which aspects of the health system are impacted by a shock. For instance, it can help understand how a pandemic significantly impacts the workforce and service delivery, consequently altering, as seen in during a pandemic or an economic crisis. A majority of the participants expressed the need for holistic frameworks that extend beyond healthcare and include non-health elements such as education, social interactions, and other components.

“*A framework that recognizes the various shock types, offers a basic core element, and can speak to the various shock types is the to be considered because it seems more responsive. This is unavoidably true in terms of the elements; it must be adaptable, capable of handling various shocks, and also agile in order to respond and react swiftly.”-* Expert in health system performance assessment

### Leadership and Governance

*Participants discussed the building blocks of the health systems (as categorized by the WHO)*. Discussions around building blocks of the health systems by WHO were discussed by the participants in the current study. Governance was discussed, in terms of traditional governance, which includes transparency, accountability, and policy-making, and also in terms of the rules that shape, how the system behaves, power structures, and trust, all of which were prominent during COVID.

“*Resilience encompasses more than just infrastructure, it also includes governance, financing, resources, information systems and services, which have an impact on the dynamics of how the health system is managed. All the elements are equally important*.”- Public Health Expert and Academician

Participants purported the importance of recognizing heterogeneity in the political perspective and health financing system. Few of them spoke about emergency insurance and gave the example of some countries which have both state and national funding for crisis situations.

## Discussion

**Fig 2:**
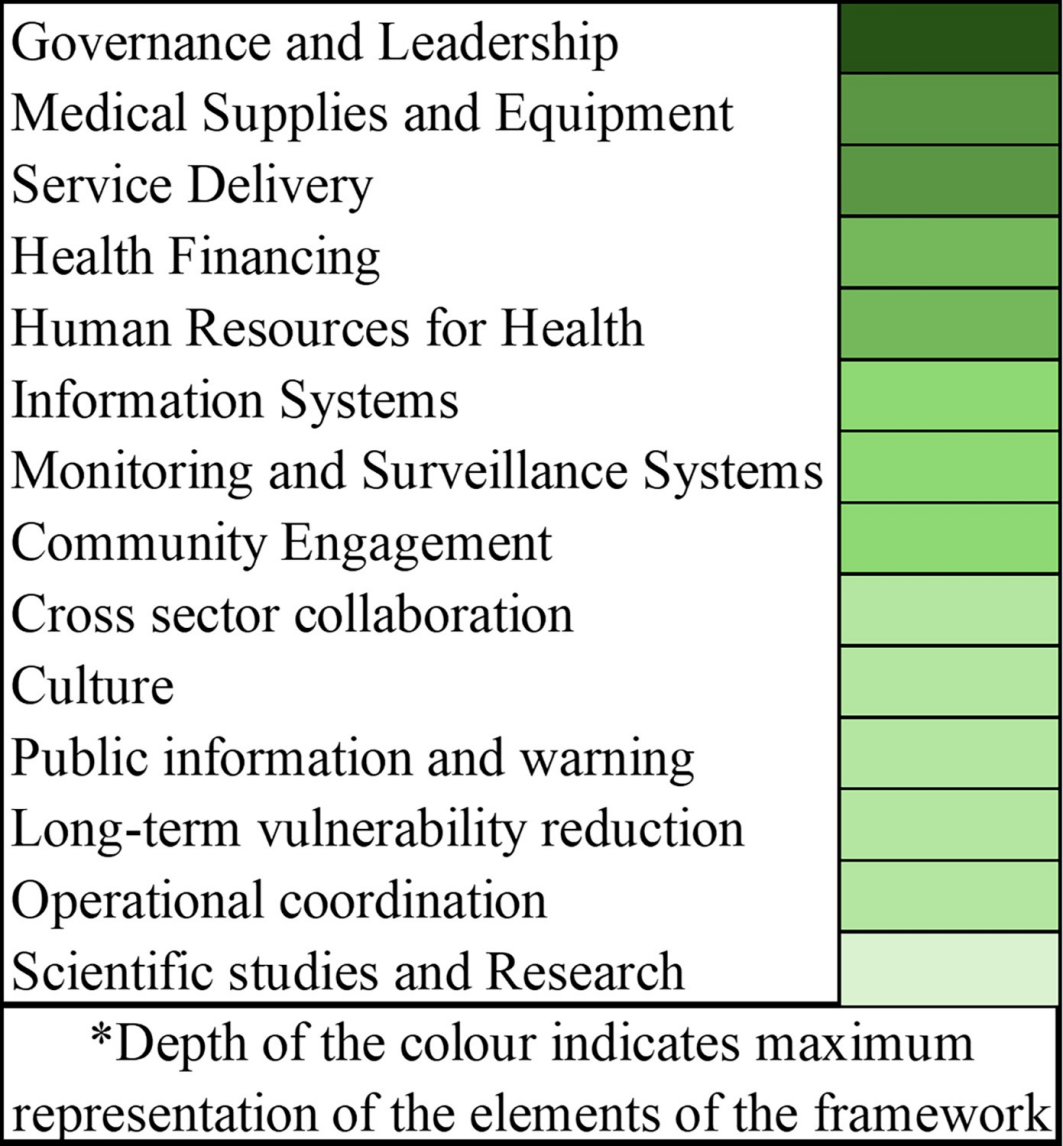
The core areas highlighted by the frameworks.

The results from this review have identified the core elements of health systems resilience in the frameworks studied (Fig 2), as well as elements that were underrepresented (and therefore with potential to bring about greater resilience).

### Governance and leadership

Governance emerges as a critical but often overlooked area for building health system resilience. The importance strengthening governance procedures, including transparency, accountability, and policy-making is highlighted in the current study. Strengthening the policies at the local level are essential for effective governance.

Effective, participatory, and forward-thinking leadership is the cornerstone of a resilient health systems.11 Weak governance systems, as exemplified by Haiti during the 2010 earthquake, often lead to humanitarian crises due to a lack of preparedness, political will, and funding. In contrast, New York City’s robust response to the 9/11 terrorist attacks was attributed to strong governance and visionary leadership [13], involving public, nonprofit, and healthcare sectors, saving lives and restoring normalcy [15] The US government’s Disaster Management Framework emphasizes the importance of identifying threats and hazards within leadership policies for disaster response [10]. The World Health Organization’s resilience framework similarly underscores the significance of strong governance and participatory leadership in assessment areas.

The current study emphasizes the importance of establishing a unified global agenda. This entails countries leveraging resources, sharing supplies, strengthening mechanisms for data sharing between nations and many more.

### Financing

The current study underscores the importance of recognizing diversity in political perspectives and health-care delivery systems. It demonstrates the importance of providing financial security to the informal, non-poor, and uninsured populations. This indicates a realization of the need for inclusive policies and financial mechanisms to promote healthcare accessibility and affordability for a greater segment of the population, particularly those who are not employed and cannot afford health insurance. The appeal for financial protection emphasizes the necessity of policies that shield individuals from catastrophic health costs while also fostering equity and inclusivity in healthcare delivery.

Financing healthcare in resource-constrained environments is complex, especially during unexpected events like pandemics. Despite the past experiences in disaster, global health financing during COVID-19 was suboptimal [16]. The UN’s Sendai framework advocates establishing public health emergency funds for all types of disasters. It emphasizes improving public financial management systems and ensuring financial accountability. This includes enhancing coordination among various governance levels (national, state, sub-state) and granting autonomy for allocating disaster response funds [17].

The WHO’s resilience framework highlights the need for flexible purchasing, financial reserves, and reallocating funds to ensure comprehensive health coverage. Meyer et al., stress the importance of adequate financing for routine and emergency health services at all facilities [12]. They recommend robust financial accounting departments and long-term financial planning. The City Resilience Framework developed by The Rockefeller Foundation underscores financial resilience beyond healthcare, considering social needs during disasters. The US Government’s Disaster Mitigation Framework emphasizes assessing long-term vulnerabilities and developing response plans to reduce the need for significant external financial assistance [10].

### Health workforce

Adequate and skilled human resources, crucial for effective response. The path to embracing a resilient health system should prioritize transitioning from a curative-centric focus to a preventive-oriented approach. A foundational step involves upskilling and mobilizing a proficient human resource capable of effectively managing health crises at the grassroots level.

To ensure an effective workforce during disasters, training in preparedness protocols and evidence-based benchmarks is crucial [12]. Routine staff, including non-medical personnel, should receive emergency preparedness training, covering infection control, protective equipment use, drills, reporting, and disaster response during normal times [4]. The UN Sendai framework calls for assessing training needs and minimizing workforce issues like absenteeism and burnout [17]. Equitable workforce distribution is vital to prevent disparities in disaster response. Health administrators play a pivotal role in rapid outbreak response and training [12]. The UN Sendai framework stresses building a strong workforce for disaster recovery, enhancing community health worker capacity, and expanding health training institutions [17].

### Service delivery

The current study emphasizes the need for mechanisms to easily repurpose infrastructure and deploy resources in times of crisis. There should be local manufacturing during emergencies, such as producing ventilators and personal protective equipment (PPE). During disasters, maintaining routine healthcare services is challenging but crucial. Key elements include preserving health facility integrity, maintain the quality of care, drug access, and minimizing supply chain disruptions [4]. Regular risk assessments and adherence to building codes are vital, requiring a risk management guide [18]. Primary care facilities play a pivotal role in integrated surveillance.

### Health information systems

Ensuring real-time information and situational awareness is vital for enhancing system resilience. This necessitates a robust IT infrastructure and the protection of information, which is particularly susceptible to cybersecurity threats.

Robust IT infrastructure aids in data management and surveillance [18]. The e-Resilience framework emphasizes ongoing ICT-based risk identification, need for cybersecurity and regulatory frameworks to ensure that the flow of information during a pandemic is never disrupted [14]. COVID-19 resulted in an unprecedented demand for digital health solutions. Successful solutions included population screening, infection tracking, resource prioritization, and targeted response design [19].

### Access to essential medicines

The current study emphasizes the need to have a surplus capacity of resources to overcome the crisis. To ensure the provision of high-quality healthcare during disasters and public health crises, a continuous supply of medical products is vital. The COVID-19 pandemic highlighted the consequences of medical supply shortages, with many nations unable to protect their staff and patients [20]. Expediting approvals for medical countermeasures was challenging for most of countries [21]. In large crises like COVID-19, international collaboration through procurement agreements (e.g., EU’s Joint Procurement Agreement) and cross-border medical care becomes crucial to avert a systemic collapse [11].

### The areas under-represented by the frameworks

The existing frameworks often do not adequately discuss few critical elements of both internal and external to the Health Systems. like information systems, monitoring and surveillance, cross-sector collaboration, cultural factors, research and scientific studies, vulnerability reduction, operational coordination, community engagement, biosecurity and biosafety, and social protection which can collectively enhance system’s ability to respond effectively to future pandemics while minimizing their impact on the individuals, community as well as society as a whole.

**Areas under-represented in the frameworks**

**Monitoring and Surveillance Systems**—Strengthening monitoring and surveillance is clearly within the purview of the health system, but it has far-reaching implications in terms of delivering an effective response strategy and accountability in the larger context [22]. While recognizing the importance of data sharing, the current study emphasizes the necessity of a bidirectional approach and underscores the crucial role of feedback mechanisms. It calls for clarity in data flow and roles and responsibilities, highlighting the imperative to construct a more effective surveillance system.

**Cross-sector Collaboration**–The current study stresses the need for established structures and guidelines for preparedness and response, involving multi-sectoral representation, including the private sector. Increased collaboration among various actors, such as policymakers, public health workers, civil society organizations, the private sector, veterinarians, academics, and religious and community leaders helps to build community resilience in the face of a pandemic. Collaborative health emergency preparedness is a common term for this approach [23].

**Culture**–The response to disaster is also driven by the culture. The dimensions of culture demonstrate how power distance affects compliance with guidelines, how uncertainty avoidance influences resistance to restrictions, how individualism-collectivism shapes cooperation, how masculinity-femininity relates to values in crisis response, and how short-term vs. long-term orientation affects adaptability during the pandemic [24].

**Scientific studies and research**–Most of the success during the COVID-19 pandemic response was due to the rapid development of vaccines and therapeutics, including innovative mRNA technologies. However, open access and sharing of real-time virus samples; clinical trials and other research data and tools; and genomic sequencing were frequently lacking [25].

**Long-term vulnerability reduction**–Long-term Vulnerability Reduction is a capability that entails several actions that reduce vulnerability. It necessitates a dedication to long-term planning and investment processes in order to ensure community resilience and vitality following a disaster. Reduced long-term vulnerabilities, combined with continuity of operations and disaster recovery planning prior to a disaster, increase resilience and the likelihood that communities and organizations will be able to perform essential functions and deliver capabilities after a disaster. As a result, the community is safer and less reliant on outside funds [10].

**Operational coordination**–Operational coordination is required to incorporate mitigation efforts into daily routines, as well as to respond to and recover from disasters. Operational coordination is not just an important component of disaster response but also aids in achieving successful mitigation. The operational coordination capability is essential for building whole-community resilience and is a prerequisite for all other mitigation capabilities.^10^ Moreover, building effective communication and coordination mechanisms from higher government to local level is essential which could yield building trust and transparency.

**Community engagement**–Community engagement is defined “*as the process by which communities and partners collaborate to build resilience through collaborative action*, *shared capacity building*, *and the formation of strong relationships based on mutual trust and respect*”. Community engagement is an essential component of disaster risk reduction and resilience building.^26^ Understanding cultural norms and social practices is considered essential for effective community involvement during health crises.

Source: Author’s compilation [10,22–26].

### Conclusion

Health system resilience reflects its ability to respond to new threats and stresses, including biological threats. Resilience is linked to vulnerability, considering environmental, cultural, and contextual factors. It is important to define the elements of a health system to understand what makes it resilient. As the field of health system resilience progresses, there’s a recognition that coherence among different frameworks will be essential for practical applications and understanding the concept. The complexity of measuring resilience is acknowledged, with a call for a broader perspective that goes beyond healthcare-centric approach to resilience and incorporating social determinants of health into assessment frameworks. These factors include economic impacts, livelihood, and individual well-being. The current study calls for the need to develop global, regional, and national frameworks to guide policies.

### Future implications of the study

This study delved into various disaster and pandemic frameworks developed as a response to catastrophic events, shedding light on their evolution, relevance, and application. Study results indicate a need for a comprehensive resilience framework, addressing both highlighted and overlooked aspects. Furthermore, there’s a call for deeper insights into diverse countries’ framework adoption and implementation to bolster swift and efficient disaster response within health systems.

A clear definition of health systems resilience is crucial, serving as the foundation for identifying assessment indicators. Policymakers must gauge health systems resilience to enhance crisis readiness and response. The capacity to navigate, control, and lead health systems during shocks is pivotal in today’s unpredictable landscape.

Initially, the framework was designed with multilevel components, encompassing international perspectives such as International Health Regulations, Sustainable Development Goals, Health status goals, Standards and Guidelines, International aid, and International collaboration. This scope broadened to include national-level policies, incorporating areas like Human Resources for Health (HRH), digital health, commodities, disaster management, research and development, service delivery planning, quality standards, and standard treatment guidelines. The framework extended further to subnational/provincial and local levels, converging towards a central theme of "health for all." At the subnational level, emphasis was placed on adaptation, adoption, and implementation, fund mobilization, purchasing, capacity building, resource mapping, and data processing. At the local level, prime focus is on data collection, service delivery, local governance, Information, Education, and Communication (IEC), and efficient resource utilization. These components collectively exert influence on the overall health of the population.

The components of the framework have consistently evolved through expert validations and peer-to-peer meetings to ensure thorough scrutiny and refinement. This has led to a development of a comprehensive framework and the components of the health system resilience framework, include provisioning, supplying, financing, and governance, and people’s health at the centre. The framework also includes the cross-cutting components of information, communication (local to central level), and relationships (across institutions). A set of indicators have been developed pertaining to each component of this framework which will be utilized in assessing the health system resilience of the countries at the local, provisional and country level. Ultimately, this study serves as a foundational step towards enhancing global health systems resilience and fostering a better-prepared response to unforeseen challenges.

## Supporting information

S1 Data Interview summaries. (XLSX)

## References

[1] Knuth K. The Term “Resilience” Is Everywhere—But What Does It Really Mean? [Internet]. 2019 [cited 2021 Dec 13]. Available from: https://ensia.com/articles/what-is-resilience/.

[2] Grimm PY, Oliver S, Merten S, Han WW, Wyss K. Enhancing the Understanding of Resilience in Health Systems of Low- and Middle-Income Countries: A Qualitative Evidence Synthesis. Int J Health Policy Manag. 2022 Jul 1;11(7):899–911. doi: 10.34172/ijhpm.2020.261 33619924 PMC9808204

[3] Haldane V, Ong SE, Chuah FLH, Legido-Quigley H. Health systems resilience: meaningful construct or catchphrase? Lancet Lond Engl. 2017 Apr 15;389(10078):1513. doi: 10.1016/S0140-6736(17)30946-7 28422019 PMC7133569

[4] Olu O. Resilient Health System As Conceptual Framework for Strengthening Public Health Disaster Risk Management: An African Viewpoint. Front Public Health. 2017;5:263. doi: 10.3389/fpubh.2017.00263 29034230 PMC5625001

[5] Ling EJ, Larson E, Macauley RJ, Kodl Y, VanDeBogert B, Baawo S, et al. Beyond the crisis: did the Ebola epidemic improve resilience of Liberia’s health system? Health Policy Plan. 2017 Nov 1;32(suppl_3):iii40–7. doi: 10.1093/heapol/czx109 . 29149311

[6] Kieny MP, Evans DB, Schmets G, Kadandale S. Health-system resilience: reflections on the Ebola crisis in western Africa. Bull World Health Organ. 2014 Dec 1;92(12):850. doi: 10.2471/BLT.14.149278 25552765 PMC4264399

[7] Houlihan CF, Youkee D, Brown CS. Novel surveillance methods for the control of Ebola virus disease. Int Health. 2017 Jun;9(3):139–41. doi: 10.1093/inthealth/ihx010 . 28582554

[8] El Bcheraoui C, Weishaar H, Pozo-Martin F, Hanefeld J. Assessing COVID-19 through the lens of health systems’ preparedness: time for a change. Glob Health. 2020 Nov 19;16(1):112. doi: 10.1186/s12992-020-00645-5 ; PMCID: PMC7675393. 33213482 PMC7675393

[9] Building health systems resilience for universal health coverage and health security during the COVID-19 pandemic and beyond: WHO position paper. Geneva: World Health Organization; 2021.

[10] National Mitigation Framework [Internet]. 2016. Available from: https://www.fema.gov/sites/default/files/2020-04/National_Mitigation_Framework2nd_june2016.pdf.

[11] Thomas S, Sagan A, Larkin J, Cylus J, Figueras J, Karanikolos M. Strengthening health systems resilience: Key concepts and strategies [Internet]. Copenhagen (Denmark): European Observatory on Health Systems and Policies; 2020. 32716618

[12] Meyer D, Bishai D, Ravi SJ, Rashid H, Mahmood SS, Toner E, et al. A checklist to improve health system resilience to infectious disease outbreaks and natural hazards. BMJ Glob Health. 2020 Aug;5(8):e002429. doi: 10.1136/bmjgh-2020-002429 32759184 PMC7409956

[13] Bhoite S, Birtill K, Cook S, Diaz S, Evans V, Fernandez A, et al. City Resilience Framework [Internet]. Arup International Development; 2015 [cited 2021 Dec 22]. Available from: https://www.rockefellerfoundation.org/wp-content/uploads/City-Resilience-Framework-2015.pdf.

[14] Karazhanova A, Barkin M, Dyakonova E. Understanding E-resilience for pandemic recovery in Asia and the Pacific [Internet]. Economic and Social Commission for Asia and the Pacific (ESCAP); 2020. Available from: https://repository.unescap.org/rest/bitstreams/e2555817-b58a-4782-a67a-92e4af5c7937/retrieve.

[15] Klitzman S, Freudenberg N. Implications of the World Trade Center attack for the public health and health care infrastructures. Am J Public Health. 2003 Mar;93(3):400–6. doi: 10.2105/ajph.93.3.400 12604481 PMC1447752

[16] Strengthening health systems during a pandemic: The role of development finance [Internet]. Organisation for Economic Co-operation and Development; 2020. Available from: https://www.oecd.org/coronavirus/policy-responses/strengthening-health-systems-during-a-pandemic-the-role-of-development-finance-f762bf1c/.

[17] Sendai Framework for Disaster Risk Reduction 2015–2030 [Internet]. 2015. Available from: https://www.preventionweb.net/files/43291_sendaiframeworkfordrren.pdf?_gl=1*1tf1mqy*_ga*MTEzNzcxMTM0NC4xNzAxNzY2MzAx*_ga_D8G5WXP6YM*MTcwMTc2ODkxNC4xLjAuMTcwMTc2OTMxOS4wLjAuMA.

[18] Joint external evaluation tool: International Health Regulations (2005). World Health Organization; 2016.

[19] Digital technology for COVID-19 response [Internet]. World Health Organization. 2020. Available from: https://www.who.int/news/item/03-04-2020-digital-technology-for-covid-19-response.

[20] International Social Security Association (ISSA) [Internet]. Building more resilient health systems. [cited 2021 Dec 2]. Available from: https://ww1.issa.int/analysis/building-more-resilient-health-systems.

[21] Pandey K. World unprepared for future pandemics: Global Health Security Index 2021. Down To Earth [Internet]. 2021 Sep 12; Available from: https://www.downtoearth.org.in/news/world/world-unprepared-for-future-pandemics-global-health-security-index-2021-80611.

[22] Sagan A, Webb E, Azzopardi-Muscat N, de la Mata I, McKee M, Figueras J. Health systems resilience during COVID-19: lessons for building back better [Internet]. Copenhagen: World Health Organization. Regional Office for Europe; 2021. (Health Policy Series: 56;). Available from: https://iris.who.int/handle/10665/348493. 37023237

[23] Akenroye TO, Abubakre A, Elbaz J, Vishnu CR, Beka Be Nguema JN, Rana G, et al. Modeling the barriers to multistakeholder collaboration for COVID-19 pandemic response: Evidence from Sub-Saharan Africa. Int Public Manag J. 2022 Mar 7;25(2):192–216.

[24] Wale H. Corporate Finance Institute [Internet]. Hofstede’s Cultural Dimensions Theory. 2021. Available from: https://corporatefinanceinstitute.com/resources/management/hofstedes-cultural-dimensions-theory/.

[25] Gostin LO, Halabi SF, Klock KA. An International Agreement on Pandemic Prevention and Preparedness. JAMA. 2021 Oct 5;326(13):1257–8. doffii: doi: 10.1001/jama.2021.16104 . 34524388

[26] Australian Institute for Disaster Resilience. Community Engagement for Disaster Resilience [Internet]. 1st ed. Australian Institute for Disaster Resilience; 2020. Available from: https://knowledge.aidr.org.au/media/7989/aidr_handbookcollection_communityengagementfordisasterresilience_2020.pdf.

